# Interspecific competition delays recovery of *Daphnia* spp. populations from pesticide stress

**DOI:** 10.1007/s10646-012-0857-8

**Published:** 2012-02-05

**Authors:** Saskia Knillmann, Nathalie C. Stampfli, Yury A. Noskov, Mikhail A. Beketov, Matthias Liess

**Affiliations:** 1 Department of System Ecotoxicology, Helmholtz Centre for Environmental Research, UFZ, Permoserstrasse 15, 04318 Leipzig, Germany; 2 Department of Ecosystem Analysis, Institute for Environmental Research, RWTH Aachen University, Worringerweg 1, 52074 Aachen, Germany; 3 Quantitative Landscape Ecology, Institute for Environmental Sciences, University of Koblenz-Landau, Fortstraße 7, 76829 Landau, Germany; 4 Institute of Systematics and Ecology of Animals, ISEA, Frunze Street 11, 630091 Novosibirsk, Russia

**Keywords:** Recovery, Competition, Toxicant, *Daphnia*, Community context, Indirect effects

## Abstract

Xenobiotics alter the balance of competition between species and induce shifts in community composition. However, little is known about how these alterations affect the recovery of sensitive taxa. We exposed zooplankton communities to esfenvalerate (0.03, 0.3, and 3 μg/L) in outdoor microcosms and investigated the long-term effects on populations of *Daphnia* spp. To cover a broad and realistic range of environmental conditions, we established 96 microcosms with different treatments of shading and periodic harvesting. Populations of *Daphnia* spp. decreased in abundance for more than 8 weeks after contamination at 0.3 and 3 μg/L esfenvalerate. The period required for recovery at 0.3 and 3 μg/L was more than eight and three times longer, respectively, than the recovery period that was predicted on the basis of the life cycle of *Daphnia* spp. without considering the environmental context. We found that the recovery of sensitive *Daphnia* spp. populations depended on the initial pesticide survival and the related increase of less sensitive, competing taxa. We assert that this increase in the abundance of competing species, as well as sub-lethal effects of esfenvalerate, caused the unexpectedly prolonged effects of esfenvalerate on populations of *Daphnia* spp. We conclude that assessing biotic interactions is essential to understand and hence predict the effects and recovery from toxicant stress in communities.

## Introduction

To evaluate the ecological effect of toxicants, the magnitude of their short-term effects and the duration of recovery for affected populations must be assessed. Models that predict the time required for recovery are often based on population growth rates (PGRs) that are obtained from analyses of single species conducted in a laboratory under optimal conditions (Barnthouse 2004). According to the approach used by Barnthouse, organisms are assumed to recover within one generation time. At the community level, the recovery of the species in abundance was found to be related to the generation time within aquatic ecosystems after general disturbance (Niemi et al. 1990) and pesticide exposure (Liess and von der Ohe 2005).

However, in several cases, the actual recovery time in such test systems, or in the field, was found to be considerably longer than one generation time. For example, the generation time of short-living cladocerans rarely exceeds 4 weeks at a water temperature of 15°C, according to a review by Gillooly (2000). Nonetheless, populations of *Daphnia galeata* were still affected by the insecticide chlorpyrifos more than 11 weeks after contamination in an outdoor test system under Mediterranean climate conditions (López-Mancisidor et al. 2008). In addition, Brock et al. (2000) reviewed studies on semi-field systems where the recovery of cladocerans that were subjected to a single exposure to organophosphorous insecticides took longer than 8 weeks after contamination. It is worth noting here that the half-life for the dissipation of chlorpyrifos and other investigated organophosphates in the water only ranges from 1–2 days (López-Mancisidor et al. 2008; Van Wijngaarden et al. 2005; Tanner and Knuth 1995). The recovery of sensitive long-living freshwater organisms is expected to take even longer and it has been found in the field that sensitive species with a generation time of 4 months or longer have not fully recovered even 1 year after exposure to toxicants (Liess and von der Ohe 2005).

In the field, more parameters affect the recovery of sensitive organisms than the generation time and growth rates identified under optimal laboratory conditions. Here, biotic and abiotic conditions as well as the ability to recolonize within the ecosystem further determine time for recovery (Liess and von der Ohe 2005; Caquet et al. 2007; Schäfer et al. 2007). Organisms in the field are often exposed to unfavourable natural conditions that can lead to reductions in the fitness and growth of individuals, such as for example competition (Hülsmann 2001), predation (Black and Dodson 1990; Hanazato 1991), salinity stress (Baillieul et al. 1996) or unfavourable pH values (Thomsen and Friberg 2002). These environmental stressors increase the effect of toxicants as shown in the review by Heugens et al. (2001).

Toxicants are also known to indirectly alter predator–prey and herbivore–producer interactions and interspecific competition (Relyea and Hoverman 2006; Fleeger et al. 2003). Considering especially changes in interspecific competition, only a few studies have linked indirect effects with a prolonged recovery. One example where such a link has been suggested was for the ecological effects of the oil spill from the Exxon Valdez in Alaska in 1989. An initial direct decline in rockweed (*Fucus gardneri*) at the shoreline caused an increase in ephemeral algae and opportunistic barnacles. In turn, these increases might have contributed to prolong the recovery period of rockweed and thereby also the recovery of associated invertebrates, as reviewed by Peterson (2001) and Peterson et al. (2003). Another example is a study on lake acidification where sensitive zooplankton species did not recover until 1–6 years after the pH of the lake had been restored to control conditions. It was assumed that the recovery of species sensitive to acidification was delayed by competition from acid-resistant species (Frost et al. 2006).

However, to our knowledge, no direct connection has been established between increases in the abundance of less sensitive species and the delayed recovery of sensitive populations in a community context under conditions that closely resemble those in the field. The aim of the study described herein was to investigate the effects of a pyrethroid pesticide on daphnids in outdoor microcosms. By doing so we also investigated the relevance of indirect effects for the recovery of organisms from toxicants under different environmental conditions.

## Materials and methods

### General

We established pond communities with variations in biotic and abiotic conditions that mirrored those found in the field. This was accomplished by the use of four different treatments that combined harvesting and the shading of communities: “Shading/Harvesting”, “No Shading/Harvesting”, “No Shading/No Harvesting” and “Shading/No Harvesting”. The treatments were designed to produce subtle effects on the biotic and abiotic conditions in the pond communities.

In the present study, we focused on genera from the family Daphniidae with different sensitivities to esfenvalerate (sensitive and insensitive D.). Long-term effects of three concentrations of esfenvalerate on populations of sensitive and insensitive D. were investigated for a period of 59 days after contamination. Changes in the structure and sensitivity of the whole communities are presented in the publication by Stampfli et al. (2011), in which only the treatments “No Shading/Harvesting”, “No Shading/No Harvesting” and “Shading/No Harvesting” were considered, as they represent a gradient of food availability and competition strength.

### Microcosms: artificial pond systems

Ninety-six outdoor microcosms were installed at the Helmholtz Centre for Environmental Research in Leipzig, Germany (51°21′13 N, 12°25′55 E). For every concentration and treatment of shading and harvesting, six replicate microcosms were established (*n* = 24 per level of concentration). Each microcosm had a volume of 80 L and was filled with 60 L of water (tap water seeded with 1 L of natural pond water). The microcosms were maintained at this volume over the course of the experiment. Communities of freshwater zooplankton and sediment were collected from five different natural ponds within a radius of 15 km from the institute and established in the microcosms at the end of May and beginning of June 2008. The natural pond sediment was mixed at a ratio of 1:1 with sand and distributed on the bottom of each tank to a thickness of approximately 1 cm. Furthermore, approximately 10 g of shredded leaves (*Populus* spp.) were added to the microcosms. The collected organisms were distributed equally among all microcosms.

Awnings were positioned close to each pond at an angle of 45° so that the microcosms were shaded at around noon each day (12–4 p.m.). All microcosms were shaded for 4 weeks until 4 days before contamination to enable comparable communities to develop in all ponds. In microcosms subjected to harvesting, biotic interaction was reduced by removing 30% of the entire pond community each week using a net (10 × 12 cm, 250-μm mesh size). Organisms were harvested from 2 weeks before contamination and continued until the end of the experiment in September 2008. The harvesting was started 10 days before the removal of the awning for the “No Shading” treatments because we assumed that more time would be required for the invertebrates to adapt to the reduction in biotic interaction than for algal growth to adapt to the increase in light.

### Pesticide exposure

Esfenvalerate, (αS)-α-cyano-3-phenoxybenzyl (2S)-2-(4-chlorophenyl)-3-methylbutyrate, is a synthetic pyrethroid that is widely used in agriculture and is highly toxic to aquatic insects and crustaceans. We used the commercial formula Sumicidin Alpha EC (BASF, Limburgerhof, Germany), which is an emulsifiable concentrate that contains 50 g/L of the active ingredient, esfenvalerate. On 4 July 2008, the microcosms were contaminated with three different concentrations (0.03, 0.3, and 3 μg/L) of the pesticide. The concentration of esfenvalerate decreased rapidly during the first hours in all setups. In addition, no significant differences in exposure among the different conditions of shading or harvesting were detected (for details, see Stampfli et al. 2011).

### Biological sampling and environmental parameters

To determine species distributions and abundances, pelagic biological samples were collected and identified over the experimental period at the following time points: 13 and 5 days before contamination (mean: 9 days), and 4, 11, 16, 44, and 59 days after contamination. The samples were collected with a sampling tube (PVC, length = 31.7 cm, radius = 3.55 cm). The lid of the sampling tube was placed first in the centre of each pond on top of the sediment. Before the tube was fitted onto the lid, the water was stirred gently in order to obtain a homogeneous distribution of organisms in the pond. Afterwards, the water from the tube (which contained 1.7% by volume of the water from the pond), including any organisms, was passed through a sieve (180 μm mesh size). The organisms obtained in this manner were preserved in 70% ethanol, identified to the level of genus (Cladocera, Chaoboridae, Culicidae, Baetidae), order (Odonata, Copepoda) or class (Ostracoda, Arachnida) and counted under a microscope. The taxonomic groups that were relatively common in the pond communities are listed in Table 1.

**Table 1:** Abundances of main invertebrate taxa in the communities without pesticide exposure. The untransformed abundances are displayed with the mean and standard deviation from 9 days before until 59 days after contamination

Taxon	Abundances (Ind./L)
*Daphnia* spp.	56 ± 60.3
Other genera of Daphniidae	131 ± 128.6
Chydoridae	54.7 ± 140
Copepoda	29 ± 37.7
Ostracoda	12.8 ± 18.7
Baetidae	1 ± 2.1
Culicidae	1.3 ± 2.3
Chaoboridae	2.8 ± 2.9
Odonata	0.05 ± 0.3

Water temperature was recorded continuously with Handylog DK501-PL data loggers (Driessen & Kern, Bad Bramstedt, Germany). Differences in UV A + UV B radiation among the treatments were measured over the course of a sunny and a cloudy day in July with a UV meter (UV–VIS radiometer RM-21, Dr. Gröbel UV-Elektronik GmbH, Ettlingen, Germany). The presence of the awning reduced the radiation at the surface of the microcosms (average daily reduction due to the awning: 76% on both a sunny and a cloudy day). Water temperature also differed between the shaded and unshaded microcosms from the time at which the awning was removed until the last sampling point (minimum daily difference = −0.6°C, maximum daily difference = −3.3°C).

To monitor water quality in the different treatments, additional parameters were measured on a weekly basis for a subsample of 32 microcosms over the entire observation period. The additional parameters included the concentration of oxygen (WTW Multi 340i meter; WTW Instruments, Weilheim, Germany), pH (HI-98127; Hanna Instruments, Woonsocket, USA), electrical conductivity (HI-98312; Hanna Instruments, Woonsocket, USA), and the concentration of chlorophyll a as a measure of algal density (relative fluorescence units—RFU; GEMINI XPS Fluorescence Microplate Reader; Molecular Devices, Sunnyvale, USA). No differences in chlorophyll a concentrations were observed between shaded and unshaded ponds. However, in unshaded ponds oxygen concentration and pH were significantly higher (mean +23.8% and +3.5%, respectively) and electrical conductivity decreased (mean −6.8%). On the basis of these measurements of physicochemical parameters, we assume that shading has an indirect effect on algal growth (Anderson et al. 1994; Falkowski and Raven 2007).

### Acute toxicity testing of esfenvalerate

Acute toxicity tests were performed to generate most comparable information on toxicological sensitivity of the Daphniidae present in the microcosms. The following species were tested: *Daphnia longispina*, *Daphnia pulex*, *Ceriodaphnia reticulata* and *Simocephalus vetulus*. The detected LC50 (96 h) values for the investigated species were similar to those previously published (Beketov 2004; Lozano et al. 1992; Werner et al. 2002). Not enough individuals of *Scapholeberis* sp. could be found for a toxicity test. For this reason we used the only existing literature value of LC50 (96 h) = 0.84 μg/L for esfenvalerate (Noskov 2011) to classify the genera.

Individuals of *D. longispina*, *D. pulex*, *C. reticulata*, and *S. vetulus* were collected in permanent and temporary ponds from the floodplains of the River Elbe, near Rosslau, Germany (51°53′06 N, 12°15′55 E), in June 2009. The organisms from the field were adapted to laboratory conditions in natural pond water under a constant air temperature of 20°C for 24 h before contamination with esfenvalerate. The pond water was passed through filter paper (mesh size: 1–2.5 nm) before the organisms were added for the toxicity tests. The electrical conductivity (EC) and pH of the used pond water were measured (HI-98312 and HI-98127; Hanna Instruments, Woonsocket, USA) and are provided in Table 2.

**Table 2:** LC50 values after 96 h with confidence intervals (CI) for the tested species and physicochemical parameters of the medium used

Species	LC50 (μg/L) with CI	Physicochemical parameters	
		pH	EC (μS/cm)
*Daphnia pulex*	0.02 (0.01–0.04)	8.12	597
*Daphnia longispina*	0.15 (0.10–0.23)	7.9	604
*Ceriodaphnia reticulata*	0.44 (0.27–0.71)	7.91	610
*Simocephalus vetulus*	2.5 (1.86–3.07)	8.15	580

For the acute toxicity tests with esfenvalerate, we applied the following concentrations: 0, 0.003, 0.01, 0.03, 0.1, 0.3, 1, and 3 μg/L. Ten replicates per control and per concentration of esfenvalerate were used. Individuals were each kept in a volume of 50 mL of medium (pond water, described above) and monitored every 24 h until 96 h after contamination. After 24 h of exposure, the medium for all test samples and controls was changed to fresh uncontaminated medium. The LC50 after 96 h was calculated using the Trimmed Spearman–Karber method (Trimmed Spearman–Karber program, version 1.5, Hamilton et al. 1977).

### Statistical analysis

The group of insensitive D. was generated by adding up the count data for all single genera in the family Daphniidae that were classified as insensitive taxa. Counted individuals and group data were fourth-root transformed, as suggested for skewed abundance data (Quinn and Keough 2002). Abundances of sensitive and insensitive D. were pooled for all treatments. Differences in mean abundance (*n* = 24 per concentration and control) at the various time points among the different concentrations of toxicant and the control were investigated with analysis of variance (ANOVA). The ANOVA was followed by pairwise *t*-tests for multiple comparisons and adjusted if the variances of the groups were not homogeneous. In the case of non-normally distributed samples, the Kruskal–Wallis test for nonparametric data was applied, followed by a nonparametric multiple-comparison test (R-package *pgirmess*, function *kruskalmc*; Siegel and Castellan 1988).

The influence of pesticide-related survival, 2 weeks after contamination and treatment of shading and harvesting, on the abundances of sensitive D. at the end of the experiment (6 and 8 weeks after contamination) was investigated with an analysis of covariance (ANCOVA). The pesticide-related survival was calculated as the ratio of the mean abundance from the samplings after contamination (11 and 16 days after contamination) to the mean abundance before contamination (−9 days) for each microcosm. Treatment was used as a categorical variable and pesticide survival of sensitive D. as a continuous variable. The models were simplified and validated in accordance with the work of Crawley (2007), by stepwise removal of nonsignificant terms until the minimal adequate model was reached.

Relations between abundances of sensitive and insensitive D. were tested for significance based on Pearson’s product-moment correlation for normally distributed data (correlation coefficient indicated with *r*) or Spearman’s rank correlation (correlation coefficient indicated with rho). Outliers were identified by checking correlations for noteworthy data points in fitted linear regression lines and applied model validation according to Crawley (2007).

We conducted a Principal Component Analysis (PCA) to assess correlations between sensitive D., insensitive D. and other taxonomic groups at pesticide concentrations with partial mortalities (0.03 and 0.3 μg/L). The selection of the linear multivariate method was based on the outcome of a preliminary Detrended Correspondence Analysis (DCA) following Leps and Smilauer (2003). The PCA was conducted and interpreted using correlation biplot scaling with centred and transformed species data (Zuur et al. 2007; Leps and Smilauer 2003). Species data were subjected to square-root transformation for reasons of most possible conformity with the previous univariate analyses. The concentration of the pesticide was log(x + 1)-transformed and added by passive ordination.

For the predicted long-term concentration–response curves we chose three abundances of insensitive D. 6 weeks after contamination, representing different percentiles of the observed abundances (“low” = 10th percentile, “medium” = 50th percentile, “high” = 90th percentile). The abundances of sensitive D. for three concentration–response curves were predicted, one for each scenario of abundance of insensitive D. The predictions on the abundance of sensitive D. at control and every concentration (displayed in % to control) were based on the regression lines that were fitted for relations between abundances of insensitive and sensitive D., 6 weeks after contamination.

Multivariate analyses were conducted using the program CANOCO 4.5 for Windows (Wageningen, Netherlands) in accordance with previous work and guides (ter Braak and Smilauer 2002; Leps and Smilauer 2003). The remaining statistical analyses and graphs were generated with R, version 2.11.1 (R Foundation for Statistical Computing, 2010).

## Results

### Taxon classification according to toxicological sensitivity

To classify taxa on the basis of their toxicological sensitivity, we determined the acute sensitivity to esfenvalerate of different genera from the family Daphniidae (Table 2). The LC50 values after 96 h of exposure for the genus *Daphnia* were found to be below the medium applied concentration of 0.3 μg/L esfenvalerate. For the other genera investigated, namely *Ceriodaphnia* and *Simocephalus*, LC50 values higher than 0.3 μg/L were found. Based on this information on toxicological sensitivity and the literature value for *Scapholeberis mucronata* (see “Acute toxicity testing of esfenvalerate” section), we divided the family Daphniidae into two groups: sensitive D. (*Daphnia* spp.) and insensitive D. (*Ceriodaphnia* spp., *Simocephalus* spp. and *Scapholeberis* spp.).

### Average population dynamics and influence of the pesticide

**Fig. 1:**
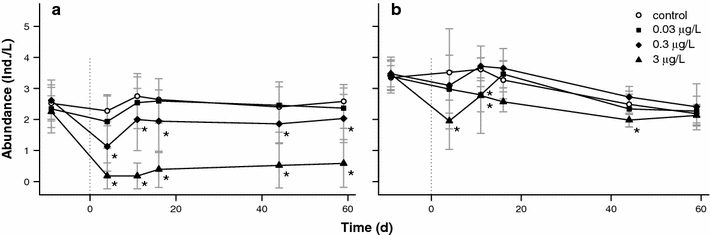
Average abundances and standard deviation of sensitive D. (**a**) and insensitive D. (**b**) for the control and the three concentrations of esfenvalerate from 9 days before until 59 days after contamination. Abundances were fourth-root transformed and averaged over all conditions of shading and harvesting. *Asterisks* indicate significant differences from the control (*p* < 0.05)

The population dynamic of sensitive and insensitive D. was observed from 9 days before contamination until 59 days after contamination for control and all concentrations of esfenvalerate (Fig. 1). The data from the treatments of shading and harvesting was pooled to analyse the general influence of the pesticide under different environmental conditions. The treatments were supposed to induce subtle changes in the environmental conditions and to increase the variability of observed abundances, which is indicated by the standard deviation in Fig. 1. The aim of only introducing subtle changes was successful, as we found no clear trends and almost no significant differences between the treatments for sensitive and insensitive D. in abundances. Only the “Shading/Harvesting” treatment showed slight differences from the other treatments for sensitive D., 6 and 8 weeks after contamination (*p* < 0.05, data not shown).

Sensitive D. presented a clear concentration–response relationship (Fig. 1a). The population size of this group was reduced significantly upon exposure to 0.3 μg/L esfenvalerate (4 days after contamination: −50.4%) and 3 μg/L (4 days after contamination: −92%) and remained reduced until the end of the experiment, more than 8 weeks after contamination. For the group of closely related but insensitive D. (Fig. 1b), no such clear concentration–response relationship was detected. Significant decreases in the size of insensitive D. populations were found only at some time points at the highest concentration of 3 μg/L (4 days after contamination: −44.5%).

To assess the prolonged recovery period of sensitive D. after pesticide exposure we conducted an ANCOVA at pesticide concentrations with partial mortality (0.03 and 0.3 μg/L, Fig. 1). We found a significant influence (*p* < 0.001) of the initial pesticide survival of sensitive D. 2 weeks after contamination on the abundance of sensitive D. 6 weeks after contamination. In contrast, for the different treatments of shading and harvesting, no significant effect was detected 6 weeks after contamination (ANCOVA, adjusted *r* ^2^ = 0.32, *df* = 43, *p* < 0.001, *n* = 48). Eight weeks after contamination, the influence of the initial pesticide survival was still significant (*p* < 0.01). Again, the treatments showed no significant influence (ANCOVA, adjusted *r* ^2^ = 0.16, *df* = 41, *p* < 0.05, *n* = 46). The ANCOVA indicated that the recovery of *Daphnia* spp. depended only on the pesticide survival at 2 weeks after contamination, when sensitive populations were lastingly affected by esfenvalerate.

### Interspecific competition between sensitive and insensitive D.

**Fig. 2:**
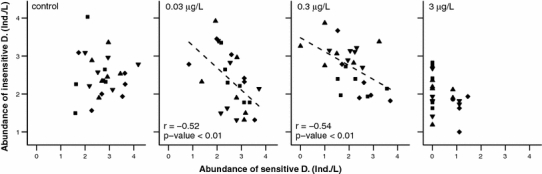
Relation between abundance of insensitive D. (6 weeks after contamination) and the abundance of sensitive D. (2 weeks after contamination) for all concentrations of esfenvalerate and treatments (*filled square* = “*Shading*/Harvesting”, *filled diamond* = “*No Shading*/Harvesting”, *filled triangle* = “*Shading*/No Harvesting”, *filled inverted triangle* = “*No Shading*/No Harvesting”). Abundances were fourth-root transformed**.** Significant correlations are represented by *r*, *p* values and fitted regression lines

To understand the observed long-term influence of initial survival to esfenvalerate on the abundance of sensitive D., we examined the interactions between sensitive and insensitive D., a competing group of closely related but less sensitive taxa. We detected indirect effects of insensitive D. when their abundance at 6 weeks after contamination was plotted as a function of the abundance of sensitive D. 2 weeks after contamination (Fig. 2). Significant negative correlations between the abundances of sensitive and insensitive D. were detected at esfenvalerate concentrations of 0.03 μg/L (*r* = −0.52) and 0.3 μg/L (*r* = −0.54). At 3 μg/L, no clear pattern was detectable owing to the limited number or absence of survivors in the sensitive D. group. In addition, no correlation between the abundances of sensitive and insensitive D. was found in the control, which indicated that interactions between the two groups only appeared when esfenvalerate was present.

**Fig. 3:**
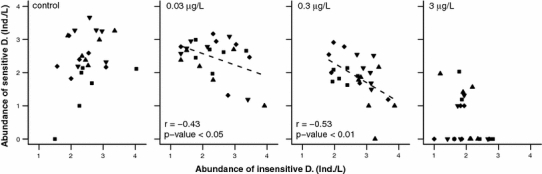
Relation between abundance of insensitive D. and the abundance of sensitive D. 6 weeks after contamination for all concentrations of esfenvalerate and treatments (*filled square* = “*Shading*/Harvesting”, *filled diamond* = “*No Shading*/Harvesting”, *filled triangle* = “*Shading*/No Harvesting”, *filled inverted triangle* = “*No Shading*/No Harvesting”). Abundances were fourth-root transformed**.** Significant correlations are represented by *r*, *p* values and fitted regression lines

After indirect positive effects of pesticide exposure on the abundance of insensitive D. had been identified, we assessed the effect of this group on the recovery of sensitive D. To do so, we plotted the abundance of sensitive D. as a function of the abundance of insensitive D. at the same time point, 6 weeks after contamination (Fig. 3). We detected a negative correlation between the abundances of sensitive and insensitive D. at 0.03 μg/L esfenvalerate (*r* = −0.43), and the correlation was even more pronounced at 0.3 μg/L esfenvalerate (*r* = −0.53). Again, no correlation between the abundances of sensitive and insensitive D. was detected at 3 μg/L esfenvalerate or in the control. The treatments had an influence on the abundances, but the observed relations were independent of the treatment (Figs. 2, 3).

The mean abundance of sensitive D. populations decreased slightly, but not significantly, at 0.03 μg/L, and significantly at 0.3 μg/L esfenvalerate, more than 8 weeks after contamination (Fig. 1a). Eight weeks after contamination, the negative correlation between the abundances of sensitive and insensitive D. was weaker than that 6 weeks after contamination, but still significant when data for both 0.03 and 0.3 μg/L esfenvalerate were combined (data not shown, Spearman’s rho = −0.38, *p* < 0.05, *n* = 33).

### Influence of other associated invertebrate taxa on sensitive D.

**Fig. 4:**
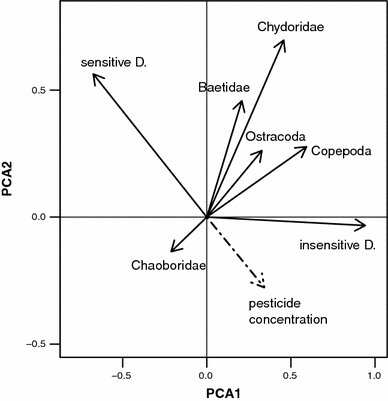
PCA correlation biplot for the relations between species data of the microcosms and pesticide concentration, 6 weeks after contamination. Only data at concentrations with partial mortality (0.03 and 0.3 μg/L) were included

We also analysed possible interactions of sensitive D. with other invertebrate taxa (8 taxon groups in total) 6 weeks after contamination using PCA. Data for pesticide concentrations with partial mortality of sensitive D. (0.03 and 0.3 μg/L, *n* = 48, Fig. 1a) was included. PCA1 explained 50.8%, and PCA2 accounted for a further 15.8% of the variation in the species data. The first four PCA axes together explained 91.4% of the observed variation. Following the interpretation of the correlation biplot diagram for relations between species (Fig. 4), insensitive D. are positively correlated with PCA1 and negatively related with sensitive D. Besides the insensitive D., none of the other taxon groups seemed to show a negative relation with sensitive D. Considering pesticide concentration, sensitive D. decreased in abundance with increasing pesticide concentration, whereas insensitive species abundances were positively correlated with pesticide concentration.

### Concentration–response curves for sensitive D. according to interspecific competition

**Fig. 5:**
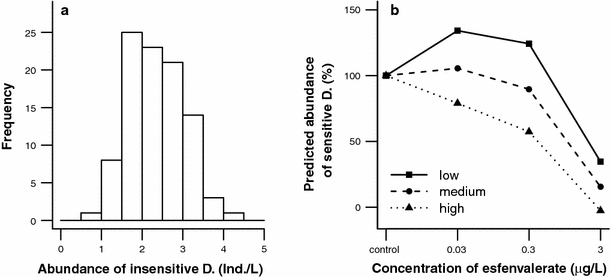
Distribution of abundances of insensitive D. (fourth-root transformed) with observed frequencies 6 weeks after contamination (**a**) (*n* = 96) and concentration–response curves for abundance of sensitive D. at three different densities of insensitive D. (“low”: 10th percentile = 1.6 Ind./L; “medium”: 50th percentile = 2.3 Ind./L; “high”: 90th percentile = 3.2 Ind./L) (**b**). Predicted abundance data for sensitive D. in % (relative to control) is based on linear models for the relations between sensitive and insensitive D., 6 weeks after contamination (Fig. 3). For the control and 3 μg/L esfenvalerate, no significant correlations were found. Here a fitted trendline was used for prediction of the concentration–response curves

On the basis of the result that the abundance of insensitive D. determined the recovery of sensitive D., we predicted concentration–response curves for sensitive D. at three abundances of insensitive D. Abundances that were assigned as “low” (1.6 Ind./L), “medium” (2.3 Ind./L), and “high” (3.2 Ind./L) were chosen to represent the 10th, 50th, and 90th percentiles of the abundances of insensitive D. (Fig. 5a). The predicted concentration–response curves revealed that, with a low level of competitors, populations of sensitive D. only showed reduced abundances at 3 μg/L esfenvalerate. In contrast, in high competitor presence, the abundance of sensitive D. was already affected slightly at 0.03 μg/L esfenvalerate, which is two orders of magnitude below the effective concentration at low levels of interspecific competition (Fig. 5b). Furthermore, the shape of the concentration–response curve at high levels of interspecific competition was flatter than that for low interspecific competition.

## Discussion

### Generation time can only be used as a relative measure of time to recovery

Taxa of the Daphniidae family responded to esfenvalerate in accordance with their toxicological classification into sensitive and insensitive D. The abundances of sensitive D. were significantly reduced at concentrations of 0.3 and 3 μg/L until the end of the experiment, whereas insensitive D. were only affected at 3 μg/L esfenvalerate at some time points during the experiment.

The generation times of organisms have proved to be important for predicting the relative recovery time of aquatic communities in mesocosms (Sherratt et al. 1999; Beketov et al. 2008) and in the field (Liess and von der Ohe 2005; Niemi et al. 1990). However, in the current study, the actual recovery times differed from the recovery time that was predicted in the model by Barnthouse (2004) on the basis of generation times. According to this model, populations of sensitive D. should have recovered in abundance within 7 days after an initial reduction of 50% (0.3 μg/L) or within 16 days after an initial reduction of more than 90% (3 μg/L) upon exposure to the toxicant. Thus, in our study, the recovery times of the populations of sensitive D. were at least eight times longer than expected at 0.3 μg/L esfenvalerate and three times longer than expected at 3 μg/L. Similar prolonged recovery times were also observed in previous studies on the effects of pesticide in the field (Liess and von der Ohe 2005) and in test systems with complex communities (Brock et al. 2000; López-Mancisidor et al. 2008).

When the time required for the recovery of sensitive populations was compared with the time derived for the recovery of the community by principal response curves (PRC) and redundancy analysis (RDA) using the dataset presented here, differences from controls were only detected up to 16 days after contamination at 0.3 μg/L (Stampfli et al. 2011). The reason for this apparent difference in effects is that multivariate analyses such as PRC or RDA are based on the structure of the entire community. Due to the dominating presence of species in the study, that were not affected by the pesticide on the long-term, these analyses probably detected other results than observed for sensitive D. alone.

### Interspecific competition delays the recovery of sensitive species

Experiments at the population level have shown that the exposure to toxicants can reduce competition and increase the abundance and survival rate of surviving conspecifics (Moe et al. 2002; Postma et al. 1994; Beketov and Liess 2005; Liess 2002). However, we assert that within communities surviving individuals of sensitive species do not benefit from increased resources after a disturbance if less sensitive and fast developing taxa are present. An increase in the abundance of less sensitive species following a reduction in the abundance of sensitive taxa has been observed in many studies (Friberg-Jensen et al. 2003; Roessink et al. 2005; Gustafsson et al. 2010; Lopez-Mancisidor et al. 2008) and reviewed by Relyea and Hoverman (2006) and Fleeger et al. (2003). In addition, sub-lethal effects of the toxicants can also lower the profit from resources of affected individuals, as already suggested in a review by Forbes et al. (2001). Esfenvalerate/fenvalerate are known to reduce filtration rates (Day and Kaushik 1987) and the fecundity of daphnids (Reynaldi et al. 2006) or mayflies (Beketov and Liess 2005). In the present study, no negative interactions between sensitive and insensitive D. were detected in the control. In contrast, upon exposure to concentrations of pesticide that caused partial mortality, negative interactions between sensitive and insensitive D. were found at densities of individuals that were comparable to those in the control conditions. These results indicate that survivors of sensitive D. might have been weakened by esfenvalerate, which probably increased the indirect effects on interspecific interaction.

We did not only observe an increase in the abundance of insensitive taxa after exposure to the toxicant, but also determined that the amount of less sensitive organisms was correlated with long-term effects on sensitive D. under all treatments of shading and harvesting. By quantifying the influence of insensitive D. on the recovery of sensitive D., we determined that the abundance of sensitive populations can change by a factor of up to 100 depending on the abundance of competitors. Multivariate statistical analyses showed that other taxonomic groups did not interact with sensitive D. as strongly as competitors that were closely related to the species, namely insensitive D. This finding is related to the concept that interspecific competition is higher for closely related taxa that use similar niches and resources.

To date, only a few studies have linked indirect effects of toxicants on field communities with the delayed recovery of sensitive species, for example, as shown for the recovery of rockweed after an oil spill (Peterson 2001). At the population level, a similar delay in the recovery of population structure due to the lack of resources has been revealed. Liess et al. (2006) investigated populations of *D. magna* and found that, after a short-term pesticide disturbance, while recovery in terms of abundance took a few days, the size structure of the populations only approached that of the control after 2 months. It was argued that the rapid development of small individuals after exposure to pesticide consumed all available resources and interrupted the long-term growth of large individuals. This hypothesis was confirmed later (Liess and Foit 2010) and a further very recent multispecies study has shown that the recovery in abundance of *D. magna* from fenvalerate is delayed by a high level of interspecific competition with mosquito larvae, which are less sensitive (Foit et al. 2012). To the best of our knowledge, this multispecies system under laboratory conditions is unique in proving a direct connection between indirect effects of pesticides and the delayed recovery of sensitive species.

### A high number of replicates facilitates the identification of recovery processes

As already mentioned, an explicit link between interspecific competition and recovery of complex communities was previously not established. This might be because the number of replicates within community test systems (e.g., microcosms, mesocosms) is restricted by the fact that these systems are very cost and labour-intensive. As an example, we selected all the studies from the review by Fleeger et al. (2003) that showed decreases and increases in the abundance of different taxa after exposure to toxicants in aquatic test systems. These reviewed experimental studies employed an average of three replicates per concentration. In contrast, we were able to use 24 microcosms for each concentration of toxicant, which enabled us to identify factors that could explain the variance in the recovery of sensitive D.

## Conclusion

The results of the study reveal that the persistence of disturbance in terms of population density by a pesticide depends strongly on the strength of interspecific competition when resource limitation is present. Given that competition is prevalent in natural communities, these biotic interactions need to be considered when predicting the recovery of affected populations. For species with a long life cycle in particular, the time needed to recover from a disturbance might reach several years or even decades if recovery is prolonged by a factor of three to eight. These findings are of crucial relevance for the risk assessment of toxicants as within the respective frameworks the duration of recovery is a relevant parameter for acceptability of effect (i.e., the EU regulation on plant protection products, EU 1107/2009).
